# The p.R249W Mutation in *LMNA*-Related Congenital Muscular Dystrophy Causes Nuclear Deformities and an Enrichment in Lamin A/C at the Ends of the Nucleus

**DOI:** 10.3390/cells15141275

**Published:** 2026-07-16

**Authors:** Catherine Harvey, Zixuan Zhu, Iden Han, Kan Cao

**Affiliations:** Department of Cell Biology and Molecular Genetics, College of Computer, Mathematical, and Natural Sciences, University of Maryland, College Park, MD 20742, USA; charvey6@umd.edu (C.H.); zzhu1910@terpmail.umd.edu (Z.Z.); ihan12@terpmail.umd.edu (I.H.)

**Keywords:** *LMNA*-related Congenital Muscular Dystrophy, laminopathies, muscular dystrophy, Lamin A/C, rare disease

## Abstract

*LMNA*-related congenital muscular dystrophy (L-CMD) is a rare genetic disorder that causes skeletal muscle weakening and wasting. Although L-CMD is caused by a variety of *de novo* point mutations in the *LMNA* gene, the p.R249W (Arg.249Trp.) pathogenic variant is the focus of this study because it is the most prevalent one among patients. We investigated the relationship between the p.R249W variant and the development of disease cellular phenotypes. We generated lentiviruses to separate p.R249W Lamin A/C localization from wild-type Lamin A/C localization, enabling us to examine how these proteins affect each other. We also developed an antibody specific for p.R249W Lamin A/C. Not only did we validate previous cellular phenotypes such as nuclear elongation, but we also identified a novel cellular phenotype. We observed overall Lamin A/C enrichment at the ends of nucleus in p.R249W patient cells. Our findings also suggest that wild-type Lamin A/C may recruit p.R249W Lamin A/C to the nuclear membrane, revealing molecular insights into the development of this dominant negative disease.

## 1. Introduction

Laminopathies are a category of rare genetic disorders caused by mutations in the *LMNA* gene. This gene encodes A-type nuclear intermediate filaments, Lamin A and Lamin C (Lamin A/C), which dimerize, self-assemble into polymers, and form the nuclear lamina [1,2]. The nuclear lamina is important for maintaining the structure and shape of the nucleus and for facilitating the localization of nuclear envelope transmembrane (NET) proteins [3]. In addition, Lamin A/C proteins function as regulators of the cell cycle, gene expression, and DNA repair [3]. Some laminopathies affect specific types of tissues, such as lipodystrophies and muscle dystrophies, while other laminopathies can affect multiple systems, such as Hutchinson–Gilford Progeria syndrome (HGPS) [4].

*LMNA*-related congenital muscular dystrophy (L-CMD) is a laminopathy that has been identified more recently than other skeletal muscle laminopathies [5]. It has similar general characterizations, such as extreme muscle hypotonia and muscle atrophy [5]. However, it is differentiated from other muscular laminopathies by the onset of its phenotypes [5]. Better-characterized laminopathies, such as Emery–Dreifuss Muscular Dystrophy (EDMD) and Limb–Girdle Muscular Dystrophy (LGMD), are considered late-onset, as symptoms tend to appear after a patient is ~2 years old. L-CMD, on the other hand, is considered early-onset because symptoms appear earlier, roughly within the first two years of life [5,6].

Like other laminopathies, multiple *de novo* dominant negative mutations in the *LMNA* gene are associated with L-CMD. The most common mutation is a c.745C>T point mutation that results in a p.R249W amino acid substitution, while the second most common mutation is a p.K32del mutation [7]. Previous studies report an increase in overall Lamin A/C in the nucleoplasm of both p.K32del and p.R249W fibroblasts [7]. In addition, other studies indicate reduced overall Lamin A/C expression in both p.K32del and p.R249W myoblasts and in p.K32del fibroblasts [7,8,9]. However, these studies focus on overall Lamin A/C expression and do not separate wild-type Lamin A/C behavior from the mutants.

A previous clinical study compared the severity of L-CMD to other skeletal muscle laminopathies by looking at the ambulation status of patients with a variety of skeletal muscular laminopathies and pathogenic variants [6]. It found that patients who had a later age of onset for disease phenotypes were more likely to achieve independent ambulation [6]. Specifically, for L-CMD patients, it found that ~63% of patients with the p.R249W variant never achieved independent ambulation, with ~50% of total p.R249W patients losing their ambulation by the time they were 5 years old [6]. This was drastically earlier than when patients with other variants lost ambulation ~10 years later, emphasizing the severity of clinical complications for L-CMD patients compared to other skeletal muscle laminopathies [6].

Our study aims to investigate the relationship between the presence of mutant Lamin proteins and total Lamin function. We focus on the p.R249W variant because, as stated previously, it is the most common variant observed in L-CMD patients [7]. In addition, this amino acid mutation is in the rod domain of Lamin A/C, a domain previously reported to mediate Lamin A/C self-assembly [2,7,10]. Therefore, we hypothesize that this amino acid mutation inhibits the proper assembly of the nuclear lamina by disrupting Lamin–Lamin interactions.

In our work, we developed a novel antibody and GFP-tagged p.R249W Lamin A/C lentiviral constructs to distinguish the localization and function of mutant proteins from those of the wild-type. We found an increase in the presence of elongated nucleus in L-CMD fibroblasts and identified a new phenotype, which is the polar end localization of Lamin A/C. We were able to replicate these phenotypes in both fibroblasts and human skeletal myoblasts (HSkMs) by transducing p.R249W Lamin A into these cells. Our work proposes two key phenotypes in both fibroblasts and myoblasts that we hope can potentially contribute to future treatment development for patients with L-CMD.

## 2. Materials and Methods

### 2.1. Primary Cell Culture

Patient-derived p.R249W fibroblasts were obtained from the L-CMD Research Foundation (Houston, TX, USA). Control fibroblasts (#HGFDFN168; Peabody, MA, USA) were obtained from the Progeria Research Foundation. Lamin A-null and control mouse embryonic fibroblasts (MEFs) were kindly gifted by Dr. Jan Lammerding at Cornell as previously mentioned [11]. All fibroblasts were cultured in DMEM (#11965092; Gibco, ThermoFisher Scientific, Waltham, MA, USA) supplemented with 10% FBS (SeraPrime, Fort Collins, CO, USA). Primary skeletal myoblasts (HSkMs) were obtained from Gibco (#A12555; ThermoFisher Scientific, Waltham, MA, USA) and cultured in growth media consisting of Ham’s F-10 Nutrient Mix (#11550043, Gibco, ThermoFisher Scientific, Waltham, MA, USA) supplemented with 20% FBS and basic fibroblast growth factor (bFGF, 1 ng/mL; R&D Systems, Minneapolis, MN, USA). All cells were passaged at 80% confluency, and the medium was changed every other day.

### 2.2. Lentivirus Generation and Transduction

To make the fluorescence-tagged Lamin A lentiviral constructs, a lentiviral construct (Figure S1) containing GFP–Prelamin A was made as previously described [12,13]. The GFP-p.R249W Prelamin A construct was created by introducing a c.745C>T mutation into the GFP–Prelamin A lentiviral vector by site-directed mutagenesis (#E0554S; NEB, Ipswich, MA, USA). Successful mutagenesis was validated through Sanger sequencing. A GFP–Lamin C construct was generated by ligating a GFP–Lamin C cDNA fragment, amplified and purified from a vector already created in the lab, into a pHR-SIN-CSGW vector using BamHI and NotI restriction enzymes (Figure S1B). This restriction enzyme selection ensured that the fragment was inserted in the correct orientation. A GFP-p.R249W Lamin C lentivirus construct was generated using site-directed mutagenesis on the created plasmid and validated by Sanger sequencing. HEK293T cells (ATCC, #CRL-3216) were co-transfected with the lentivirus construct of interest, a pMDG.2 envelope vector, and a psPAX2 packaging vector using Fugene 6 (#E2691; Promega, Madison, WI, USA). The supernatant was collected 48 h and 72 h post-transfection and filtered through a 0.45 μm filter. The supernatant was aliquoted into 1 mL aliquots and kept at −80 °C. Primary fibroblasts and myoblasts were transduced using media containing polybrene (#sc-134220; Santa Cruz, Dallas, TX, USA) at a final concentration of 8 mg/mL. Media were replaced with fresh growth media 48 h post-transduction, and cells were maintained until a GFP signal was observed under the microscope, usually around the 72 h time point.

### 2.3. Python Program Development for Calculating Nuclear Curvature and Eccentricity

A Python (version: 3.11.6) program was written for the extraction of nucleus boundaries and for the calculation of nucleus curvatures. First, we separated each nucleus by cropping such that each cropped image contains only one nucleus. Pre-processing was then performed. Each nucleus image was binarized using Otsu’s method implemented by the threshold function in the OpenCV library. Holes in the binarized image smaller than 600 pixels squared were filled using the remove_small_holes function in the OpenCV library. Next, Gaussian smoothing was performed on the binarized images using Scikit-image’s Gaussian filter function with a sigma value of 1. The binarized images were then eroded to compensate for Gaussian smoothing with a circular kernel with a radius of 3 pixels. The morphological closing (a dilation followed by an erosion) of the binarized images was performed with the morphologyEx function in the OpenCV library using a circular kernel with a radius of 3 pixels.

The contours of the nucleus were approximated by the boundary of the binarized images after pre-processing. The Scikit-image library’s findContours function under the measure module was used to extract the boundaries of the pre-processed binary images. Since each image only contained one nucleus, the longest contour was selected and visualized on the raw image, verifying the selection of the correct contour. The contours were then smoothed using a Savitzky–Golay filter to ensure better fits to the nucleus’s circular shapes.

The curvature at each contour point was calculated with the same method previously published by our lab [14]. In the calculations, positive values were used for convex curvatures, and negative values were used for concave curvatures. The colored visualization was clipped between a max value and a min value such that the curvatures greater than the max value were visualized with the same color as the max value, and the same applies for the curvature values less than the min value. The min value was selected to be 0 as our focus is the analysis of the positive curvatures, and the max value was selected to be 0.075 /pixels. The curvature points were ordered such that the starting point is selected to be the point that is the closest to the intersection of the minor axis and the ellipse that is fitted to each nucleus. This ensured the consistent relative positional arrangement of the curvature points for each nucleus.

The eccentricity of the nucleus is defined as the eccentricity of the ellipse of best fit to the nucleus contour using the fitEllipse function in the OpenCV library.

### 2.4. Western Blot

Cell lysate samples were prepared as previously reported [15]. Samples were loaded into a 10% TGX acrylamide gel (#1610173, BioRad, San Francisco, CA, USA) and were semi-wet-transferred onto a 0.45 μm nitrocellulose membrane (#1620145, BioRad, San Francisco, CA, USA). Membranes were incubated at room temperature for 1 h in 5% milk + TBST. Membranes were then incubated with the following primary antibodies: Lamin A/C (MAB3211; Millipore Sigma, Rockville, MD, USA, 1:750 dilution), p.R249W Lamin A/C (custom, 1:250 dilution), GAPDH (sc-47724; Santa Cruz, Dallas, TX, USA, 1:500 dilution), and beta actin (A3854; Millipore Sigma, Rockville, MD, USA, 1:5000 dilution). Membranes were washed 3 × 10 min each and then incubated with the following secondary antibodies: anti-mouse (sc-516,102; Santa Cruz, Dallas, TX, USA, 1:5000) and anti-rabbit (211–035–109; Jackson Immuno-Research, West Grove, PA, USA, 1:5000). Membranes were washed 3 × 10 min and incubated in ECL substrate (Thermo cat. # 34580). The imaging of blots was done on a Biorad ChemiDoc imager (San Francisco, CA, USA).

### 2.5. Immunofluorescence and Immunofluorescence Imaging

Cells were seeded at the same density in either 4-well chamber slides or 45 mm glass-bottom dishes. Cells were washed twice with 1X PBS and fixed with 4% paraformaldehyde at room temperature for 15 min. Cells were permeabilized using 0.5% Triton buffer in PBS for 5 min at room temperature. Cells were then washed once with TBS and then incubated in 4% BSA+TBS blocking buffer for 1 h at room temperature. Cells were then incubated with primary antibody in 4% BSA+TBS overnight at 4 °C. The following primary antibodies were used: Lamin A/C antibody (MAB3211; Sigma, Rockville, MD, USA, 1:500), Lamin B1 (Santa Cruz, Dallas, TX, USA, 1:500), alpha-tubulin (sc-3293; Santa Cruz, 1:1000), and myosin heavy chain (sc-376157; Santa Cruz, Dallas, TX, USA, 1:250). Cells were washed five times with TBS and incubated in 4% BSA+TBS containing secondary antibody and Hoechst33342 for 1 h at room temperature. The following secondary antibodies and Hoechst stain were used: Alexa Fluor 488 donkey anti-mouse IgG (1:1000, Invitrogen, ThermoFisher Scientific, Waltham, MA, USA), Alexa Fluor 594 donkey anti-mouse IgG (1:1000, Invitrogen, ThermoFisher Scientific, Waltham, MA, USA), Alexa Fluor 594 donkey anti-rabbit IgG (1:1000, Invitrogen, ThermoFisher Scientific, Waltham, MA, USA), Alexa Fluor 594 donkey anti-goat IgG (1:1000, Invitrogen, ThermoFisher Scientific, Waltham, MA USA), and Hoechst 33342 (1:500 of 1 mg/mL, Invitrogen, ThermoFisher Scientific, Waltham, MA, USA). Epifluorescence images were taken using a Zeiss Axio Observer A1 Inverted Phase Contrast Fluorescence Microscope (Zeiss, Columbia, MD, USA).

### 2.6. Fluorescence Profile Plot for Confocal Imaging

Z-stack images were acquired with a Zeiss LSM710 and analyzed with FIJI (ImageJ 2.9.0). The RGB Profiler plugin was used to analyze and develop the RGB distribution plots for a given line segment along a single z-stack slice. The value given along the y-axis represents the intensity value of a fluorescent color at a given point on the line segment drawn. The x-axis represents the distance along the line segment.

### 2.7. Cell Cycle Assay

Cells were harvested using Trypsin–EDTA 0.05% (ThermoFisher Scientific, Waltham, MA, USA, #25300054) and washed once with PBS. Cells were resuspended in 70% ice-cold ethanol and incubated at 4 °C for 1 h. Cells were then washed once with PBS and treated with RNase (1 μg/μL) at room temperature for 30 min. After RNase treatment, 5 μg of propidium iodide (Invitrogen, ThermoFisher Scientific, Waltham, MA, USA) was added to the samples and incubated at 37 °C for 30 min. Samples were then kept on ice and analyzed with a FACS Canto II (BD). Data from experiments were analyzed using FlowJo v10.10.1.

### 2.8. RNA Isolation and qPCR

Cells were grown to 80% confluency and then detached with 0.05% Trypsin–EDTA (Gibco, cat# 25300054) and pelleted. Total RNA was extracted using TRIzol–chloroform extraction (Life Technologies, ThermoFisher Scientific, Waltham, MA, USA) and purified using the Qiagen RNeasy kit (Qiagen, Germantown, MD, USA, cat# 74104). The RNA concentrations of samples were determined using a NanoDrop 2000 spectrophotometer (Thermo Fisher Scientific, Waltham, MA, USA). The iScript Select cDNA Synthesis kit (Bio-Rad, San Francisco, CA, USA) was used to synthesize cDNA from 1 μg of RNA. Quantitative RT-PCR was performed on a CFX96 Real-Time PCR Detection System (C1000 Thermal Cycler, Bio-Rad) in technical triplicates using BioRad SYBR Green Supermix (Bio-Rad, San Francisco, CA, USA). The primers used are listed in Table 1:

### 2.9. Data Analysis

Statistical analysis was performed using GraphPad Prism 9 software. Data significance between the two samples was determined using an unpaired Student’s *t*-test. Experiments were performed with at least 3 biological replicates and are shown in bar graphs as the mean ± standard deviation.

## 3. Results

### 3.1. R249W-Specific Biological Tools Were Created for This Study to Separate and Investigate the Roles of p.R249W Lamins

L-CMD is a rare disease, and despite initial clinical and genetic descriptions, relatively few studies have investigated the pathogenic mechanisms of the Lamin A/C p.R249W variant. Also, few biological tools are available for specifically studying this variant. Therefore, our first step was to create fluorescence-tagged p.R249W Lamin A/C lentiviral constructs to use for our experiments (Figure S1A). cDNA fragments containing either the sequence for GFP-tagged Prelamin A or Lamin C were inserted into a lentiviral vector that has previously been described [12,13,16] (Figure S1B). Site-directed mutagenesis was utilized to create the p.R249W Lamin A/C constructs from our wild-type constructs by introducing the c.C745T point mutation into the Prelamin A and Lamin C cDNA sequences (Figure S1A). The same protocol was used to create DsRed-tagged wild-type and p.R249W Prelamin A lentiviruses [17]. This was done so that we could co-transduce Lamin A and Lamin C into the same cells and visualize their localizations separately. Once constructs were made and validated with Sanger sequencing, lentiviruses were prepared, and HEK293T cells were transduced for 72 h. Cells were then collected and lysed for Western blot analysis.

**Figure 1:**
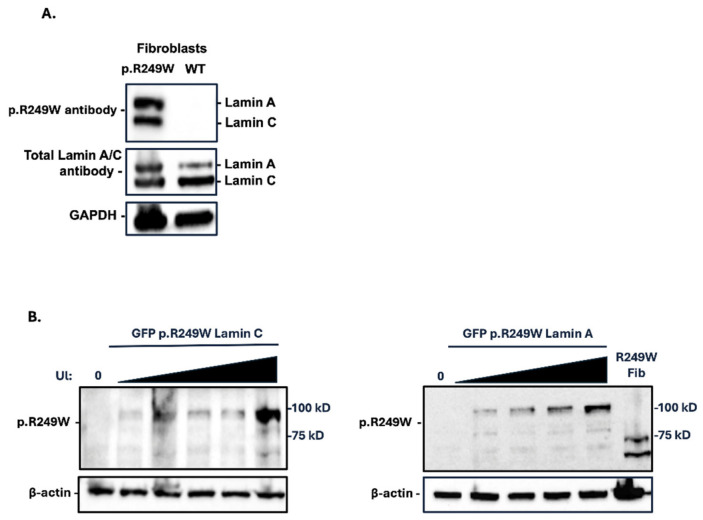
Development of p.R249W-specific antibody and lentiviruses. (**A**) Western blot analysis of primary p.R249W patient fibroblasts and wild-type fibroblasts showing specificity of custom p.R249W Lamin A/C antibody. Commercial anti-Lamin A/C antibody (MAB3211) that binds to 464–572 amino acid region of Lamin A/C was also used to demonstrate that total Lamin A/C (~73 kD and ~64 kD) is present in both samples. Anti-GAPDH antibody (~37 kD) was used for loading control. (**B**) Western blot analysis of HEK293T cells transduced with GFP-tagged p.R249W Lamin A or GFP-tagged Lamin C in increasing increments of volume of virus transduced using custom p.R249W Lamin A/C antibody. GFP-p.R249W–Lamin A should be ~100 kD in size, while GFP-p.R249W–Lamin C should be ~92 kD. Last lane shows p.R249W patient fibroblast sample to emphasize that p.R249W antibody is specific for both endogenous p.R249W Lamin A/C and GFP-tagged p.R249W Lamins. Anti-β-actin antibody was used as loading control.

We also generated a custom rabbit polyclonal anti-p.R249W antibody against the 244–254 amino acid region of p.R249W-Lamin A/C, using the same strategy previously employed to develop an anti-progerin antibody for HGPS [18]. This antibody specifically detected both p.R249W Lamin A and p.R249W Lamin C in primary fibroblasts from an L-CMD p.R249W patient on Western blots but showed no binding to wild-type Lamin A and Lamin C in control healthy fibroblasts (Figure 1A and Figure S2A). Not only did this indicate the specificity of the antibody, but it confirmed the presence of the p.R249W mutation in both Lamin A and Lamin C proteins. As expected, this antibody also detected the GFP-tagged p.R249W Lamin A and C proteins in transduced HEK293T cells but not the endogenous wild-type Lamin A/C (Figure 1B). Although our antibody was functional in Western blot assays, it was not specific in immunofluorescence applications (Figure S2B). However, this antibody allowed us to specifically detect and validate the presence of the p.R249W mutation in both Lamin A and Lamin C, which has not been previously done. It also further validated that we should investigate the effects of the p.R249W mutation in both Lamin A and Lamin C proteins, not just one or the other. With our new plasmids, we can do this by separating the p.R249W Lamins not only from wild-type Lamins but from each other.

### 3.2. p.R249W Patient-Derived Fibroblasts Exhibit Nuclear Disruption and Polar Localization of Lamin A/C to Ends of Nucleus

**Figure 2:**
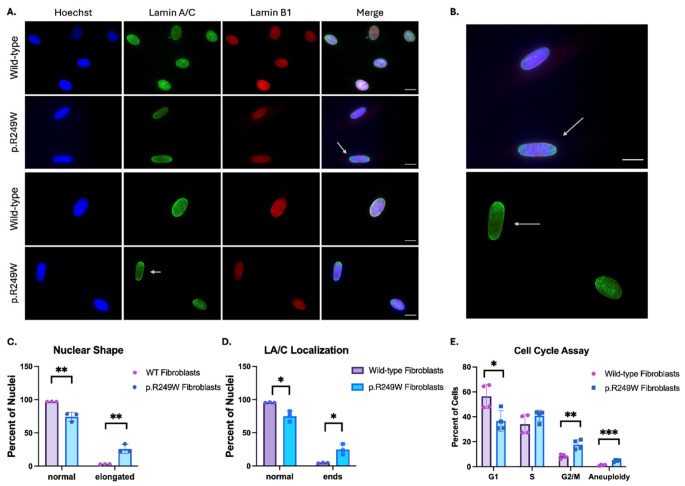
Nuclear morphology and function abnormalities are seen in patient-derived p.R249W fibroblasts. (**A**) Representative immunofluorescence images of wild-type fibroblasts and p.R249W patient-derived fibroblasts emphasizing elongation (top two panels) and Lamin A/C polar localization (bottom two panels) in p.R249W fibroblasts. Staining against Lamin A/C (green) and Lamin B1 (red) was used. (**B**) Zoom in of image from (**A**) showing example of elongated nucleus (top image) and Lamin A/C polar localization (bottom image) with white arrow. (**C**) Quantification of nucleus that were elongated. (**D**) Quantification of nucleus that exhibited polarized localization of Lamin A/C. For all counting, 500 nucleus were counted for each replicate (N = 3). Values shown as percent of population of nucleus counted. Scale bar = 10 μm. (**E**) Cell cycle analysis of fibroblasts using propidium iodide (PI) live cell staining and analysis using flow cytometry, N = 3. * indicates *p*-value ≤ 0.05; ** indicates *p*-value ≤ 0.01; *** indicates *p*-value ≤ 0.001.

To investigate the effects of p.R249W Lamins, we prepared patient-derived L-CMD p.R249W fibroblasts and control fibroblasts for an immunofluorescence analysis of nuclear phenotypes. Previous studies have reported abnormal shapes of iPSC-derived L-CMD myoblast nucleus, including nuclear blebbing and elongation [19,20]. In the primary skin fibroblasts from the L-CMD p.R249W patient, we observed that ~25.8% of p.R249W fibroblasts had an elongated nucleus, while only ~3.2% of wild-type fibroblasts had an elongated nucleus (Figure 2A–C). However, unlike other studies, we did not see any significant increase in nuclear blebbing. We also did not observe any significant changes in overall Lamin A/C gene expression nor any significantly noticeable trends between overall cell morphology between the two samples (Figure S3A–C).

However, while observing the p.R249W nucleus, we noticed another phenotype. Lamin A/C exhibited non-uniform subnuclear localization, accumulating at the elongated poles of the nuclear periphery instead of forming a continuous layer beneath the nuclear envelope (Figure 2A,B,D). We performed a randomized count of nucleus under the fluorescence microscope and found that ~24.81% of p.R249W nucleus had Lamin A/C polar localization specifically to the ends of the nucleus (Figure 2D). This was a significant increase compared to the ~2% seen in wild-type nucleus. Notably, we found that this phenotype did not correlate with the phenotype of other nuclear intermediate filaments, such as Lamin B1 (Figure S4).

In addition to nuclear morphology, we also sought to obtain a general idea of nuclear function during the cell cycle. To do this, we stained fibroblasts with propidium iodide (PI) and analyzed them by flow cytometry for a cell cycle assay (Figure 2E and Figure S3D) We found a significant increase in p.R249W fibroblasts that are arrested in the G2/M phase. In addition, we found a significant increase in aneuploidy in p.R249W fibroblasts. However, when we co-transduced HeLa cells with either wild-type or R249W Lamin A/C, we found no significant differences in localization between the two during mitotic stages (Figure S5). This data suggests that the p.R249W mutation itself is not directly affecting mutant Lamin A/C localization but may be affecting the nucleus’s ability to break down and reform properly during mitosis through protein interactions and other mechanisms.

### 3.3. p.R249W Fibroblast Nucleus Exhibit Higher Positive Curvature and Higher Eccentricity Compared to Wild-Type

**Figure 3:**
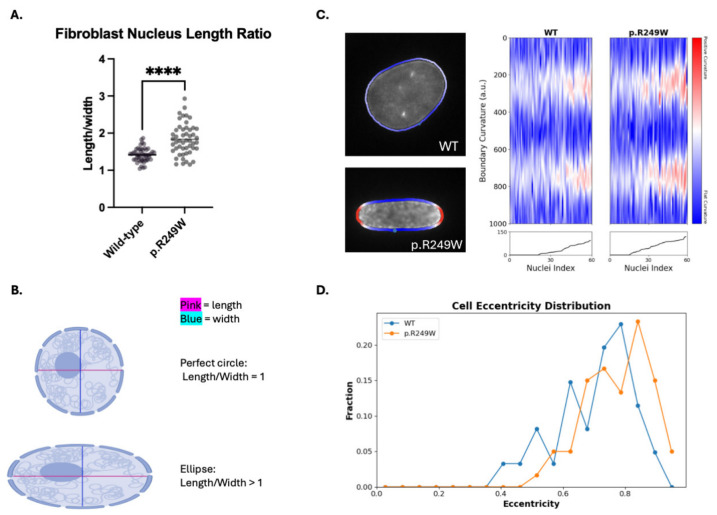
An imaging analysis of patient-derived p.R249W fibroblasts in comparison to wild-type fibroblasts. (**A**,**B**) The length and width of nucleus from both wild-type fibroblasts and p.R249W fibroblasts were measured using the line and measurement tools in FIJI. The length was then divided by the width and then plotted as a ratio value. Each point is one nucleus (N = 60 nucleus). (**C**) A custom Python program was designed and used on wild-type and p.R249W patient-derived fibroblasts to calculate the curvature value at a single point around the circumference of a nucleus. Red indicates a more positive curvature, while blue indicates no curvature (flat). The curvatures for all nucleus of each sample were linearized and arranged from the most positive curvature (right) to the least positive curvature (left) (N = 60 nucleus). (**D**) The eccentricity distributions of nucleus from wild-type fibroblasts (blue) and p.R249W patient-derived fibroblasts (orange) were graphed as a line graph, with the y-axis being the fraction of the population. A value closer to 1 indicates higher eccentricity and therefore a more elliptically shaped nucleus (N = 60 nucleus). **** indicates a *p*-value ≤ 0.0001.

To quantify the elongated nucleus, a custom Python program based off a previously published MATLAB program (v7.12.0) was created to analyze the curvature of R249W fibroblast nucleus and compare it to the wild-type [14] (Figure 3 and Figure S6). First, we measured the “lengths” of nucleus and divided it by their “widths” using FIJI software (Fiji Is Just ImageJ) (Figure 3A,B). The “length” of the nucleus was determined to be the longest end-to-end distance of a nucleus, while the “width” was determined to be the shortest. We found a significant increase in the length-to-width ratio in p.R249W fibroblasts, indicating elongated nucleus.

Then, we used the Python program to trace the circumference of each nucleus and calculate the curvature at each single point along it (Figure 3C). The color range in this program indicates high positive curvature in red, while no curvature is shown in blue (Figure 3C). The program calculated the curvatures of each nucleus in our cell populations, linearized them, and lined them up from the least to most overall positive curvature from left to right. We found that p.R249W fibroblasts exhibited higher levels of positive curvature at the ends of nucleus compared to the wild-type, indicating a more elliptical shape (Figure 3C). In addition, we found that p.R249W nucleus had much higher eccentricity than the wild-type, further supporting the fact that they are more elliptical (Figure 3D). Taken together, this data aligns with our initial observations of elongated nucleus in p.R249W patient-derived fibroblasts and validates previous reports of the same phenotype.

### 3.4. p.R249W Lamin A Polar Localization to the Ends of the Nucleus

**Figure 4:**
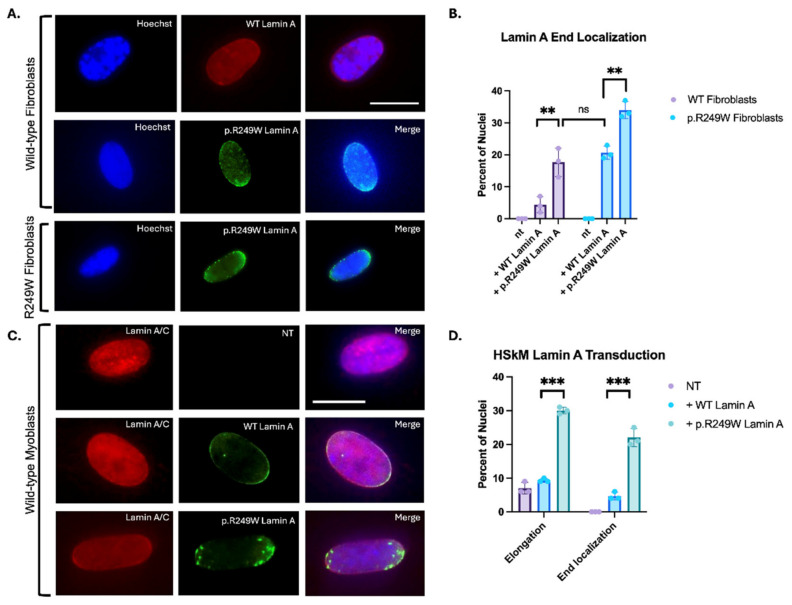
Transduction of p.R249W Lamin A reproduces elongation and abnormal localization of mutant Lamin A in wild-type fibroblasts and myoblasts. (**A**,**B**) Immunofluorescence imaging of wild-type fibroblasts and p.R249W patient-derived fibroblasts transduced with either DsRed-tagged wild-type Lamin A (red) or GFP-tagged p.R249W Lamin A lentivirus (green). End localization of fluorescence-tagged Lamin A was quantified per transduction group and expressed as percentage of nucleus. Total of 100 nucleus were counted per replicate (N = 3). (**C**,**D**) Immunofluorescence imaging of myoblasts transduced with either GFP-tagged wild-type Lamin A or p.R249W Lamin A (green). Immunostaining for overall Lamin A/C was also performed (red). Percentage of nucleus with elongation and end localization of transduced Lamin A was quantified and expressed as percentage of nucleus. Total of 100 nucleus were counted per replicate (N = 3). Scale bar: 10 μm. ns indicates non-significant *p*-value; ** indicates *p*-value ≤ 0.01; *** indicates *p*-value ≤ 0.001.

So far, we have been comparing Lamin A/C localization in samples that are not isogenic. To determine whether polar localization is due to the p.R249W mutation or due to the genetic differences between p.R249W fibroblasts and wild-type fibroblasts, we transduced both wild-type and p.R249W fibroblasts with either GFP–wild-type Lamin A/C or GFP-p.R249W Lamin A/C. This allowed us to compare the localization of p.R249W Lamin A/C to that of the wild-type in the same genetic background and determine whether the polar localization phenotype is mainly composed of mutant Lamins, wild-type Lamins, or a combination of both (Figure 4A,B and Figures S7 and S8).

Despite a previous study indicating the potential effects that GFP tags may have on Lamin–Lamin assembly [21], we chose to proceed with utilizing fluorescence-tagged Lamins because it is currently the only method that we can use to separate p.R249W Lamin A/C from the wild-type in microscopy-based assays. During our transductions, we saw that the distribution of our GFP-tagged wild-type Lamin A/C was similar to the distribution of endogenous wild-type Lamin A/C in our wild-type fibroblasts (Figure 2A and Figure 4). Therefore, we have confidence that our wild-type assays are not behaving abnormally, and our system is a good set-up for investigating the polar localization phenotype, which we have already established is a phenotype of endogenous Lamin A/C in p.R249W fibroblasts (Figure 1).

We found that when p.R249W Lamin A was transduced into wild-type fibroblasts, polar localization was observed at a significantly higher rate than in wild-type fibroblasts transduced with wild-type Lamin A. In addition, in wild-type fibroblasts that were transduced with p.R249W Lamin A, fluorescence intensity did not differ significantly between nucleus exhibiting end localization and nucleus that did not (Figure S7A). This suggests that this phenotype is not dependent on variation in the expression of the transduced plasmid.

However, when wild-type Lamin A was transduced into p.R249W fibroblasts, end localization was observed at a similar rate as when p.R249W Lamin A was transduced in wild-type fibroblasts (Figure 4B and Figure S7B). This suggests that not only does p.R249W Lamin A tend to localize to the ends of the nucleus, but it also influences wild-type Lamin A localization to the nuclear ends. Furthermore, when p.R249W Lamin A was transduced in p.R249W fibroblasts, there was a significant increase in end localization compared to all other conditions (Figure 4B). This dosage-dependent enhancement indicates that elevated levels of p.R249W Lamin A exacerbate nuclear polarization, supporting the conclusion that end-biased Lamin A localization represents a bona fide, mutation-driven phenotype of p.R249W L-CMD.

Interestingly, the transduction of wild-type fibroblasts with either wild-type or p.R249W Lamin C did not result in any significant changes in Lamin A/C subnuclear localization (Figure S8). In contrast, the transduction of wild-type Lamin C into p.R249W patient-derived fibroblasts led to the partial polarized localization of Lamin C (Figure S8). These findings indicate that p.R249W Lamin C alone is insufficient to induce the end localization phenotype. Rather, the presence of endogenous p.R249W Lamin A is sufficient to drive polarized Lamin organization, with Lamin C passively redistributing in the mutant nuclear environment. Therefore, for the remainder of this study, we focused on p.R249W Lamin A as the primary determinant of the observed nuclear polarization phenotype.

### 3.5. Presence of End Localization and Elongation Phenotypes in p.R249W Lamin A-Expressing Myoblasts

Since L-CMD primarily affects skeletal muscle development, we transduced wild-type human myoblasts with p.R249W Lamin A to determine whether its expression would also induce phenotypes (Figure 4C,D). Indeed, we found that the presence of p.R249W Lamin A increased the percentage of elongated nucleus and nucleus with Lamin A/C polar localization. This data shows that, similarly to fibroblasts, the presence of p.R249W Lamin A in myoblasts is sufficient to induce the development of L-CMD phenotypes.

### 3.6. Wild-Type Lamin A/C Is Required for Mutant p.R249W Lamin A Localization Towards the Nuclear Membrane

Together, our findings to date show that mutant p.R249W Lamin A exerts a dosage-dependent effect on nuclear elongation and polar Lamin localization, whereas p.R249W Lamin C does not measurably contribute to this phenotype. We therefore hypothesized that p.R249W Lamin A perturbs nuclear architecture through its interaction with wild-type Lamin A/C.

**Figure 5:**
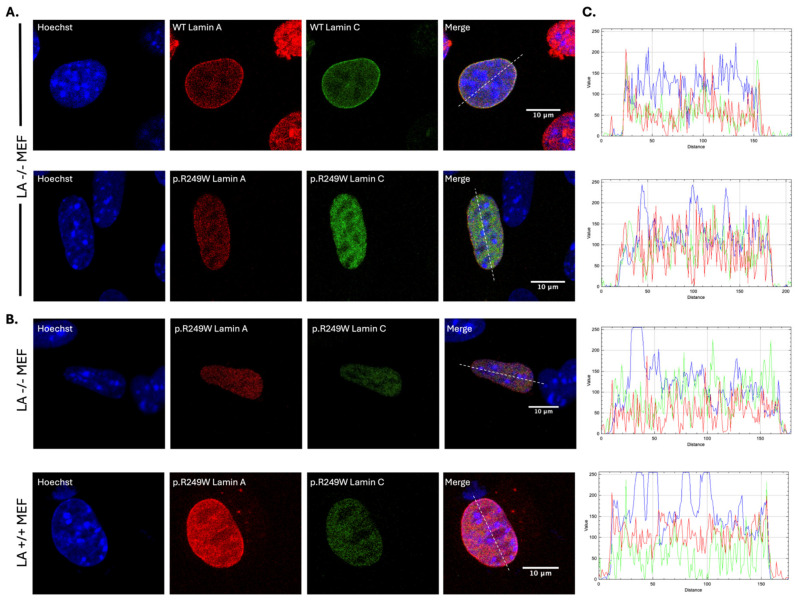
p.R249W Lamin A/C mislocalizes in absence of wild-type Lamin A/C. (**A**) Confocal fluorescence imaging of LA-/- MEF cells transduced with wild-type or p.R249W Lamin A (red) and Lamin C (green). (**B**) Confocal imaging of LA-/- MEF and LA-/- MEF cells transduced with p.R249W Lamin A (red) and Lamin C (green). Scale bar: 10 μm. (**C**) Fluorescence profiles of Lamin A (red line) and Lamin C (green line) across linear segment of nucleus (indicated by dotted white line). Nuclear boundaries are indicated by Hoechst fluorescence intensity (blue).

To test this, we next examined how mutant p.R249W Lamin A behaves in the absence of wild-type Lamin A/C. We utilized mouse embryonic fibroblasts that either contained or lacked Lamin A (LA +/+ or -/- MEFs) [11] and transduced them with either p.R249W Lamin A/C or wild-type Lamin A/C (Figure 5 and Figure S9). Unexpectedly, in LA-/- MEFs, p.R249W Lamin A/C was predominantly localized within the nucleoplasm rather than at the nuclear periphery, whereas wild-type Lamin A/C localized appropriately to the nuclear membrane (Figure 5A,C and Figure S9). Although this initially appeared surprising, it is consistent with prior observations showing increased nucleoplasmic Lamin A/C localization in L-CMD patient fibroblasts compared with wild-type controls [7]. Importantly, this phenotype is notable because the p.R249W variant retains an intact nuclear localization signal and does not appear to alter sites known to regulate Lamin targeting to the nuclear envelope. Therefore, these findings suggest that p.R249W Lamin A exhibits impaired lamina incorporation in the absence of wild-type Lamin A/C, consistent with previous reports describing increased nucleoplasmic retention of mutant inner nuclear Lamins.

In contrast, when p.R249W Lamin A/C was transduced into LA+/+ MEFs (Figure 5B,C and Figure S9), a greater proportion of the mutant protein localized to the nuclear periphery. However, this rescue effect is only partial and does not recover the phenotype to wild-type levels (Figure S9C). These findings suggest that the presence of endogenous wild-type Lamin A/C partially promotes the peripheral localization of p.R249W Lamin A/C. Thus, while p.R249W Lamin A/C displays impaired lamina incorporation on its own, interactions with wild-type Lamin A/C modestly improve its targeting to the nuclear membrane.

## 4. Discussion

L-CMD is a dominant negative laminopathy that remains severely understudied. Its main symptoms include the weakening and wasting of skeletal muscles, making patients unable to live and move independently [22]. Although several model systems (Table 2) have been used to study L-CMD, only a limited number of studies have directly interrogated the molecular mechanisms driven by the Lamin A/C p.R249W variant, leaving its disease-specific pathogenic pathways poorly defined. Our study uses p.R249W Lamin A/C-specific bio-tools to not only validate that the p.R249W mutation is present in both Lamin A and Lamin C proteins but also identify a novel phenotype in L-CMD cells and propose a relationship between p.R249W and wild-type Lamin A/C.

### 4.1. The p.R249W Mutation Disrupts the Correct Localization of Lamin A/C and Cell Cycle Progression

Based on our data, not only do p.R249W cells have more elongated nucleus (Figure 2 and Figure 3), but we also observed a previously unrecognized enrichment in overall Lamin A/C at the polar ends of nucleus in the presence of p.R249W mutant proteins (Figure 2 and Figure 4). One hypothesis is that a hydrophilic arginine (R) mutated to a hydrophobic tryptophan (W) in the exposed rod domain of Lamin A/C may be disrupting Lamin A/C assembly through biochemical or structural changes. This is supported by the previous literature indicating that the rod domain plays a major role in the assembly process [2,7,10]. The identification of these two phenotypes is important for future studies because they can be used as biomarkers when investigating mechanistic pathways and also be used as disease biomarkers for future drug screening and therapeutic development. This application has already been implemented in other laminopathies, for example, progeroid disorders, where a previous study utilized nuclear morphology as a biomarker to determine the efficacy of a drug called lonafarnib on cells from patients who had progeroid disorders [25].

In addition, our data indicates cell cycle misregulation in p.R249W fibroblasts, specifically at the G2/M phase, along with increased aneuploidy (Figure 2E). This suggests that p.R249W Lamin A/C disrupts the proper execution of mitosis, despite its ability to localize properly (Figure S5). Therefore, mitotic disruption may be caused by disruptions in p.R249W Lamin A/C’s ability to interact properly with other nuclear proteins present, such as wild-type Lamin A/C. This interpretation is consistent with the highly dynamic nature of the nuclear lamina during mitosis, which requires the coordinated disassembly and reassembly of overall Lamin A/C. The impaired assembly of Lamin A/C may hinder efficient lamina breakdown and reformation, thereby compromising mitotic fidelity. Cell division is especially critical during muscle development, as myogenic progenitors must proliferate extensively before exiting the cell cycle to differentiate and fuse into multinucleated myofibers [26]. The disruption of mitotic progression, particularly defects in nuclear lamina disassembly and reassembly, can limit progenitor expansion, impair differentiation, and compromise nuclear organization in developing muscle. Accordingly, the G2/M defects, increased aneuploidy, and altered Lamin A/C dynamics observed in p.R249W cells provide a mechanistic link between mutant Lamin-induced mitotic dysfunction and the severe muscle pathology seen in L-CMD.

### 4.2. The Presence of p.R249W Lamin A Is Sufficient to Trigger the End-Polarization Localization of Lamin A/C

Based on our data (Figure S8), we concluded that transducing only p.R249W Lamin C into wild-type cells is not sufficient to induce polarized Lamin A/C localization. We therefore focused subsequent analyses on p.R249W Lamin A, transducing it into both wild-type fibroblasts and myoblasts. In these contexts, p.R249W Lamin A consistently exhibited polarized localization (Figure 4). Notably, wild-type Lamin A also adopted a polarized distribution when transduced into p.R249W patient-derived fibroblasts (Figure 4). Importantly, increasing the dosage of p.R249W Lamin A in p.R249W patient-derived cells further enhanced the degree of polarized Lamin localization, indicating a dosage-dependent effect. Together, these findings demonstrate that the presence of p.R249W Lamin A results in the dosage-dependent polarized localization of Lamin A/C in p.R249W cells.

### 4.3. Wild-Type Lamin A/C Is Required to Target p.R249W to the Nuclear Periphery

Although our data demonstrates that mutant p.R249W Lamin A is sufficient to induce nuclear elongation and polarized Lamin A/C localization in cells containing endogenous wild-type Lamin A/C (Figure 4), this phenotype fails to fully manifest in the complete absence of wild-type Lamin A/C proteins (Figure 5). This indicates that p.R249W Lamin A does not act independently but instead requires interaction with the existing Lamin A/C network to remodel nuclear architecture. We propose that wild-type Lamin A/C provides a structural scaffold that permits the incorporation of mutant Lamin A/C into the lamina, enabling the dosage-dependent polarization and elongation phenotype; in its absence, mutant Lamin A remains largely nucleoplasmic and is unable to generate organized lamina asymmetry.

It is possible that the recruitment of p.R249W Lamin A/C to the nuclear membrane is detrimental to the cell. It has been previously reported that in p.R249W patient-derived myoblasts, there was abnormal localization of nuclear membrane proteins SUN1 and SUN2 [8], suggesting the disruption of the LINC (Linker of Nucleoskeleton and Cytoskeleton) complex. The aberrant incorporation of mutant Lamin A/C into the nuclear lamina may therefore destabilize nucleo-cytoskeletal coupling, alter force transmission, and exacerbate mechanical stress in muscle cells. Future work will be required to define how p.R249W Lamin A/C affects nucleo-cytoskeleton interactions and nuclear mechanics during both interphase and mitosis and determine whether limiting mutant Lamin incorporation into the nuclear membrane can mitigate cellular dysfunction. Elucidating these mechanisms will be critical for understanding how LMNA mutations translate nuclear architectural defects into progressive muscle degeneration and may reveal new therapeutic strategies to restore nuclear envelope integrity.

Overall, additional mechanistic studies will be essential to fully define the impact of p.R249W Lamin A/C on nuclear organization and cellular function. An analysis of LINC complex components (e.g., SUN1 and SUN2) could reveal defects in nuclear positioning during muscle differentiation, consistent with the established role of Lamin A in organizing these structures [27]. In parallel, a systematic investigation of interactions with key cell cycle regulators (pRb, Fos, and ATM), along with the validation of cell cycle phenotypes in controlled systems, will be critical, as preliminary observations suggest nuclear division abnormalities but remain limited by variability in transduction-based models. Furthermore, an evaluation of active chromatin markers may clarify the direct role of p.R249W Lamin A/C in chromatin activation and gene regulation. Although beyond the scope of this study, these directions will be important for advancing the mechanistic understanding of disease pathology.

## 5. Conclusions

Our study investigates the cellular phenotypes of L-CMD associated with the p.R249W mutation in Lamin A/C. Using a novel p.R249W-specific Lamin A/C antibody, we validated the presence of the p.R249W mutation in both Lamin A and Lamin C in patient-derived cells and confirmed expression in the lentiviral constructs used throughout this study (Figure 1). Through immunofluorescence analysis combined with a customized Python-based quantification pipeline, we confirmed nuclear elongation as an established phenotype in p.R249W patient-derived fibroblasts (Figure 2 and Figure 3). In addition, we identified a previously uncharacterized phenotype—polar end localization of Lamin A/C (Figure 2). To further investigate this phenotype, we transduced p.R249W Lamin A/C lentiviruses into fibroblasts and myoblasts to distinguish mutant from wild-type Lamin A/C localization and assess whether the phenotype could be recapitulated (Figure 4). We found that p.R249W Lamin A exhibits polar end localization and may influence the localization of wild-type Lamin A (Figure 4). In contrast, p.R249W Lamin C alone was insufficient to reproduce this phenotype (Figure S8). Finally, using MEFs with or without Lamin A, we observed that p.R249W Lamin A/C displayed increased nucleoplasmic localization in Lamin A-deficient (LA^−^/^−^) cells, whereas in Lamin A-expressing (LA^+^/^+^) cells, it preferentially localized to the nuclear envelope (Figure 5). These findings suggest a reciprocal relationship in which mutant Lamin A/C influences wild-type Lamin A/C organization, while wild-type Lamin A/C also modulates mutant protein localization.

Overall, this study provides new insight into the interplay between wild-type and p.R249W Lamin A/C and identifies two measurable phenotypes, nuclear elongation and polar end localization, which may serve as useful readouts for future therapeutic screening. Further studies will be needed to define the underlying mechanisms, including potential effects on cell cycle regulation and nuclear envelope organization.

## Figures and Tables

**Table 1:** qPCR primers.

Gene	Forward Sequence	Reverse Sequence
ACTB	CTGGAACGGTGAAGGTGACA	AAGGGACTTCCTGTAACAATGCA
Lamin A	GCAACAAGTCCAATGAGGACCA	CATGATGCTGCAGTTCTGGGGGCT CTGGAT
Lamin C	CTCAGTGACTGTGGTTGAGGA	AGTGCAGGCTCGGCCTC

**Table 2:** Representation of previous L-CMD studies that utilized different model organisms.

Author	Model
Bertrand, A.T. et al. [7]	Human and mouse primary cells
Steele-Stallard, H.B. et al. [20]	iPSCs
Walker, S.G. et al. [23]	Drosophila
Gregory, E.F. et al. [24]	C. elegans

## Data Availability

The original contributions presented in this study are included in the article/Supplementary Material. Further inquiries can be directed to the corresponding author.

## Supplementary Materials

The following supporting information can be downloaded at https://www.mdpi.com/article/10.3390/cells15141275/s1, Figure S1: Illustrations of Lamin A/C lentiviruses; Figure S2: p.R249W custom antibody design and characterization; Figure S3: Extended characterization of wild-type and p.R249W fibroblasts; Figure S4: Lamin B1 localization is not significantly correlated with polar localization of Lamin A/C or significantly mislocated between wild-type fibroblasts and R249W fibroblasts. Figure S5: Representative confocal imaging of mitotic stages of HeLa cells transduced with either fluorescence-tagged wild-type Lamin A/C or R249W Lamin A/C. Figure S6: There is no significant difference in the mean curvature and area distributions of wild-type and p.R249W patient-derived fibroblasts; Figure S7: Extended characterization of Lamin A transduction in fibroblasts; Figure S8: Transduction of p.R249W Lamin C is not sufficient to induce polar localization phenotype; Figure S9: Representative fluorescence imaging of MEF transduction with either wild-type Lamin A/C or p.R249W Lamin A/C.
