# Supplementary material for: The p.R249W Mutation in LMNA-Related Congenital Muscular Dystrophy Causes Nuclear Deformities and an Enrichment in Lamin A/C at the Ends of the Nucleus
Source: Cells. 2026 Jul 16;15(14):1275. doi: 10.3390/cells15141275 (PMC13406450; doi:10.3390/cells15141275)
Supplement: Supplementary file 1 [file cells-15-01275-s001.zip › cells-4247757-supplementary.pdf]

Supplementary Figures:

Figure S1: Illustrations of Lamin A/C lentiviruses.

Figure S2: p.R249W custom antibody design and characterization.

Figure S3: Extended characterization of wild-type and p.R249W fibroblasts.

Figure S4: Lamin B1 localization is not significantly correlated with polar localization of Lamin A/C or significantly mislocated between wild-type fibroblasts and R249W fibroblasts.

Figure S5: Representative confocal imaging of mitotic stages of HeLa cells transduced with either fluorescence-tagged wild-type Lamin A/C or R249W Lamin A/C.

Figure S6: There is no significant difference in the mean curvature and area distributions of wild-type and p.R249W patient-derived fibroblasts.

Figure S7: Extended characterization of Lamin A transduction in fibroblasts.

Figure S8: Transduction of p.R249W Lamin C is not sufficient to induce a polar localization phenotype.

Figure S9: Representative fluorescence imaging of MEF transduction with either wild-type Lamin A/C or p.R249W Lamin A/C.

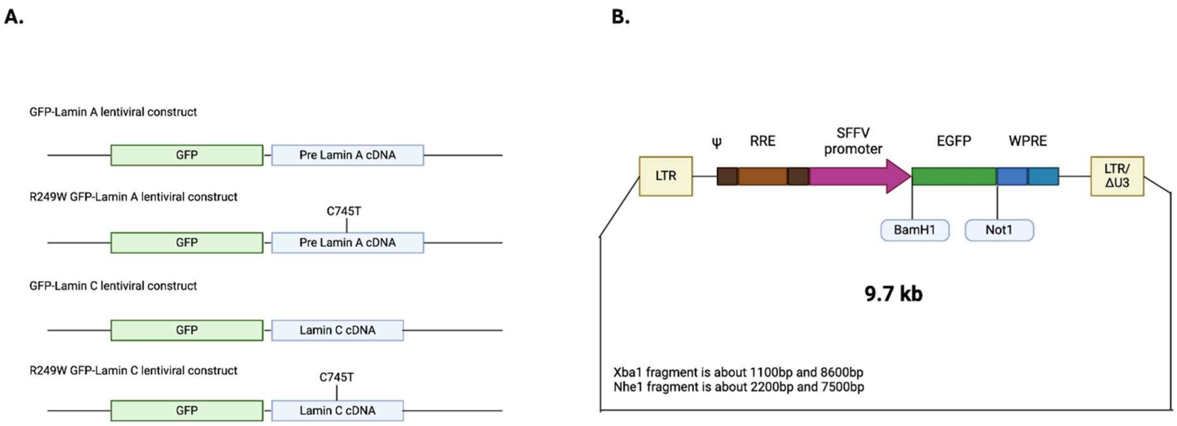

Figure S1: Illustrations of Lamin A/C lentiviruses. (**a**) Illustration of the point mutation introduced into the cDNA of Pre Lamin A and Lamin C via site-directed mutagenesis to create p.R249W mutant Lamin A/C plasmids. (**b**) Map of the lentiviral vector backbone. BamH1 and Not1 sites were used to clone the cDNA for the GFP proteins of interest in the correct orientation.

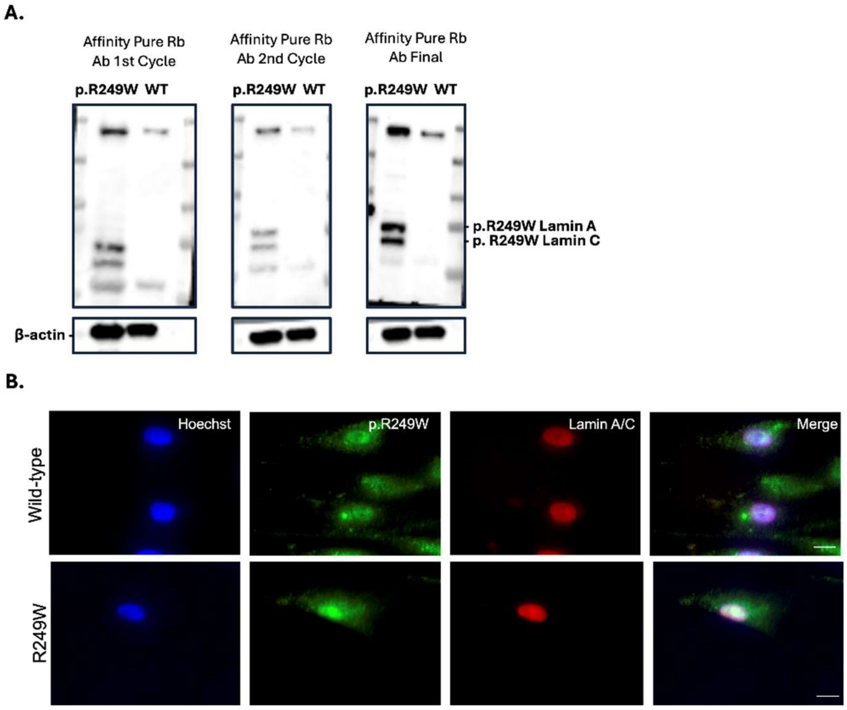

Figure S2: R249W custom antibody design and characterization. (**a**) Western blot of p.R249W and wild-type fibroblasts to assess the specificity of three antibody elution samples from a rabbit injected with the p.R249W peptide. The antibody specifically detected p.R249W Lamin A and p.R249W Lamin C at the expected molecular weights; although a higher-molecular-weight nonspecific band was observed, it did not interfere with Western blot detection or interpretation. The final Ab elution was chosen as the antibody for this study due to its p.R249W Lamin A/C specificity. An anti-β-actin antibody was used as a loading control. (**b**) Immunofluorescent images of wild-type and p.R249W patient-derived fibroblasts stained with the custom p.R249W antibody (green) against p.R249W Lamin A/C. Staining for overall Lamin A/C with a commercial antibody (red) was also performed as a control for the p.R249W staining. In immunofluorescence, the p.R249W-specific antibody also produced detectable signals in wild-type cells, likely due to the same nonspecific binding responsible for the high–molecular-weight band observed by Western blot. As a result, this antibody is not suitable for immunostaining applications. Scale bar: 10 um (N=3)

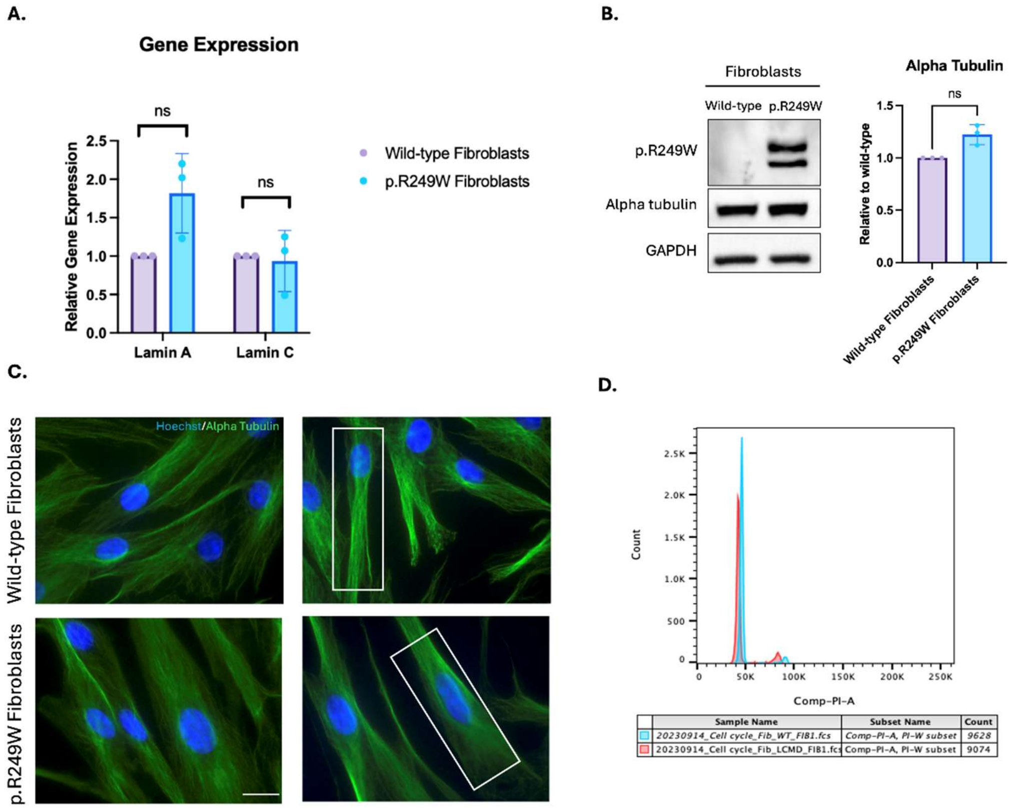

Figure S3: Extended characterization of wild-type and p.R249W fibroblasts. (**a**) Quantification of relative gene expression of Lamin A and Lamin C compared to actin in wild-type fibroblasts and p.R249W fibroblasts. Ns means not statistically significant. N=3. (**b**) Western blot of wild-type fibroblasts and p.R249W fibroblasts using our custom p.R249W antibody to validate the presence p.R249W Lamin A/C in the p.R249W fibroblast sample. An antibody specific for alpha-tubulin was also used with another antibody specific for GAPDH being used as the loading control. Relative alpha-tubulin protein expression is shown in the bar graph. N=3 (**c**) Representative immunofluorescence images of wild-type fibroblasts and p.R249W fibroblasts stained for alpha-tubulin (green). Two images are shown for each sample. White box indicates example of cells where alpha-tubulin morphology appeared similar despite nuclear shape. N=3 (**d**) Live cells were stained with propidium iodide (PI) and analyzed on a BD Canto II. PI is a DNA-binding stain, so the values on the x-axis represent the amount of DNA in a cell. The y-axis represents cell count. The first peak on the left represents the G1-phase population. The second peak represents the G2/M-phase population. The population of cells between the two peaks is the S-phase population. Values to the left of the G1 peak were considered background. Values to the right of the G2/M-phase peak were considered aneuploid. N=4

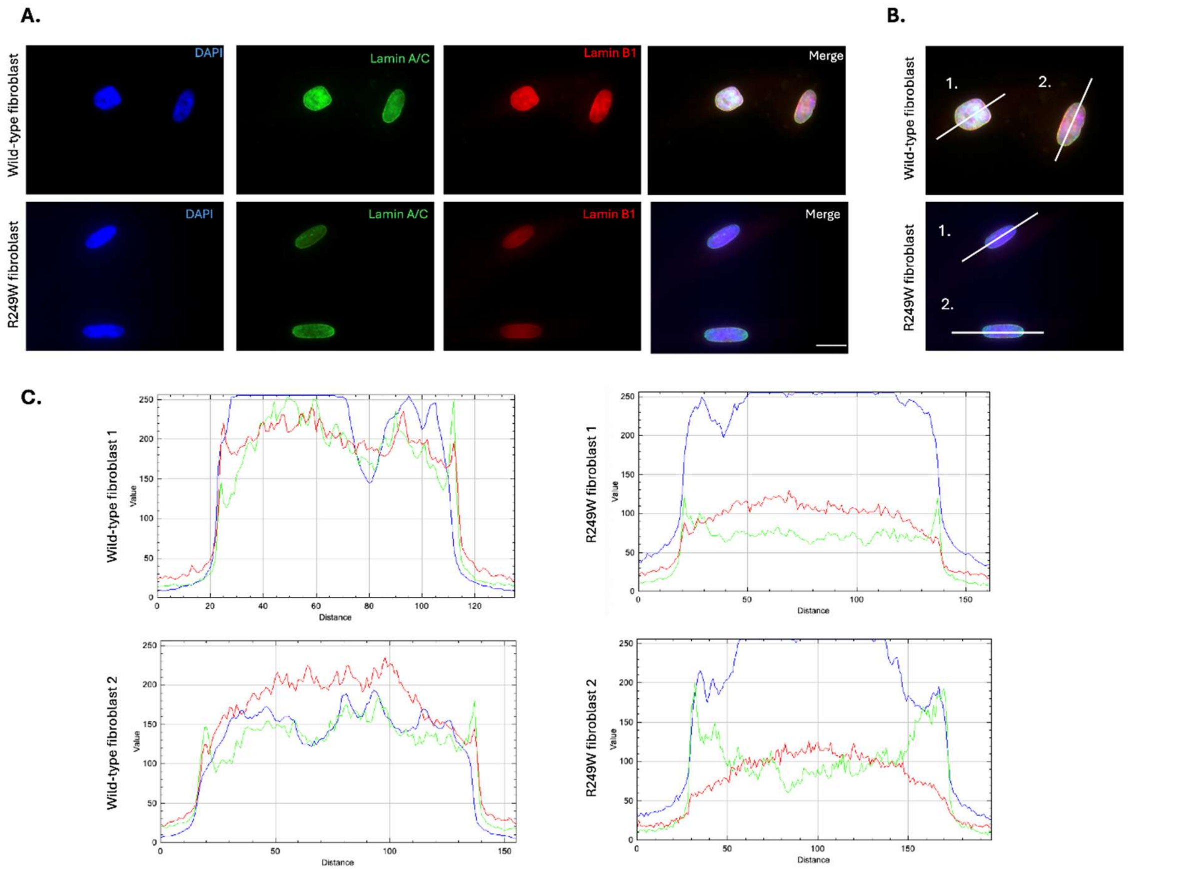

Figure S4: Lamin B1 localization is not significantly correlated with polar localization of Lamin A/C in wild-type fibroblasts and R249W fibroblasts. (**a**) Representative immunofluorescence images of nuclei from either wild-type fibroblasts or R249W fibroblasts stained for Lamin A/C (green) and Lamin B1 (red). (**b**) the line segments for (**c**) the RGB profiler plots used to show the distribution of green and red fluorescence across the drawn line of the nucleus. Scale bar: 10 μm. N=3

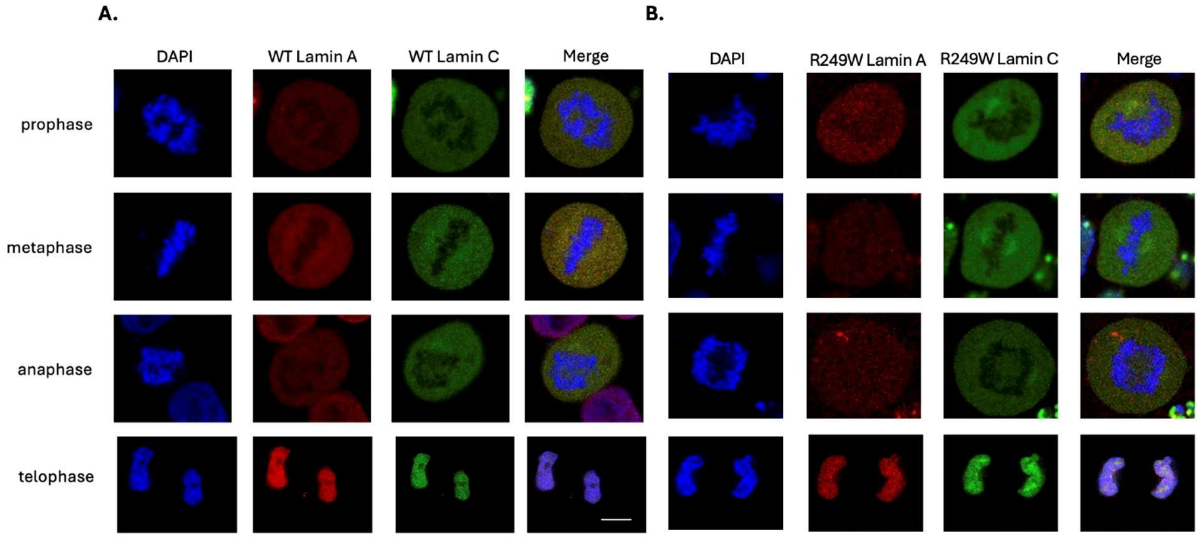

Figure S5: Representative confocal imaging of mitotic stages of HeLa cells co-transduced with either fluorescence-tagged wild-type Lamin A/C or R249W Lamin A/C. HeLa cells were co-transduced with lentiviruses for either wild-type Lamin A/C or R249W Lamin A/C. Both wild-type Lamin A and R249W Lamin A are DsRed tagged (red fluorescence). Wild-type Lamin C and R249W Lamin C are GFP-tagged (green fluorescence). DAPI is represented in blue. Representative images of N=3 replicates. Scale bar: 10 μm

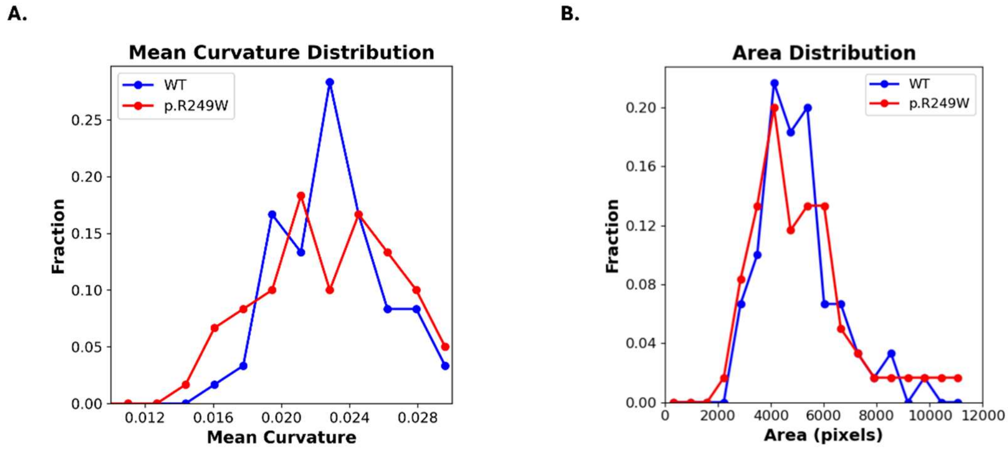

Figure S6: There is no significant difference in the mean curvature and area distributions of wild-type and p. R249W patient-derived fibroblasts. (**a**) All curvature points from one nucleus were averaged to yield a single mean value per nucleus. These values were then plotted, with mean curvature on the x-axis and the fraction of the cell population with that mean curvature on the y-axis. (**b**) The area of the nuclei was calculated and plotted, with the area of the nucleus on the x-axis and the fraction of the cell population with that area on the y-axis. The wild-type fibroblast distribution is shown in blue, and the p.R249W fibroblast distribution is shown in red. N=60 nuclei

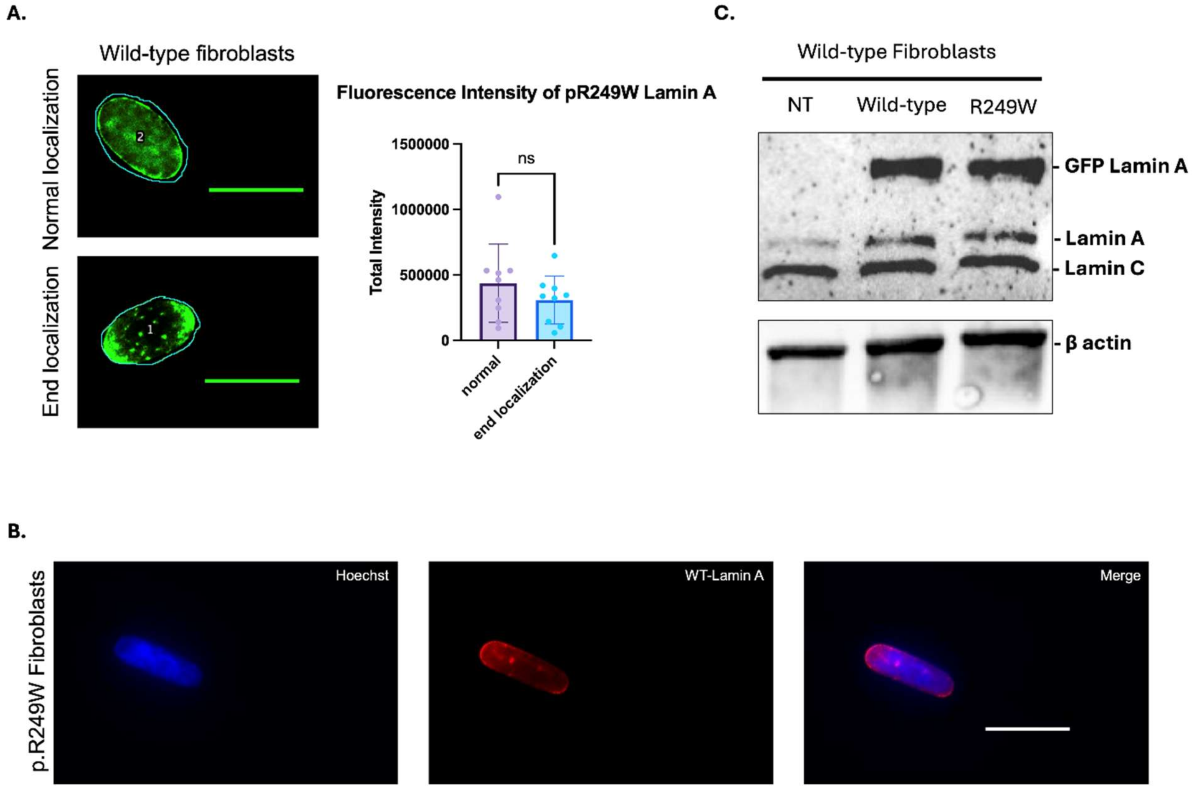

Figure S7: Extended characterization of Lamin A transduction in fibroblasts. (**a**) Immunofluorescent images of wild-type fibroblasts transduced with p.R249W Lamin A that either exhibited "normal" localization or polar end localization of p.R249W Lamin A. The exposure was kept constant across all images, and the total fluorescence intensity was measured using FIJI as the integrated density of the manually outlined nuclear area. N=10 nuclei each group and the scale bar: 10 μm. (**b**) Wild-type Lamin A, transduced into p.R249W patient-derived fibroblasts, exhibits polar localization. Immunofluorescence imaging of p.R249W patient-derived fibroblasts transduced with DsRed-tagged wild-type Lamin A. Scale bar: 10 μm (c) Western blot of wild-type fibroblasts transduced with either wild-type Lamin A or R249W Lamin A. A commercial Lamin A/C antibody (MAB3211) was used to detect total Lamin A/C. An anti-β-actin antibody was used as a loading control.

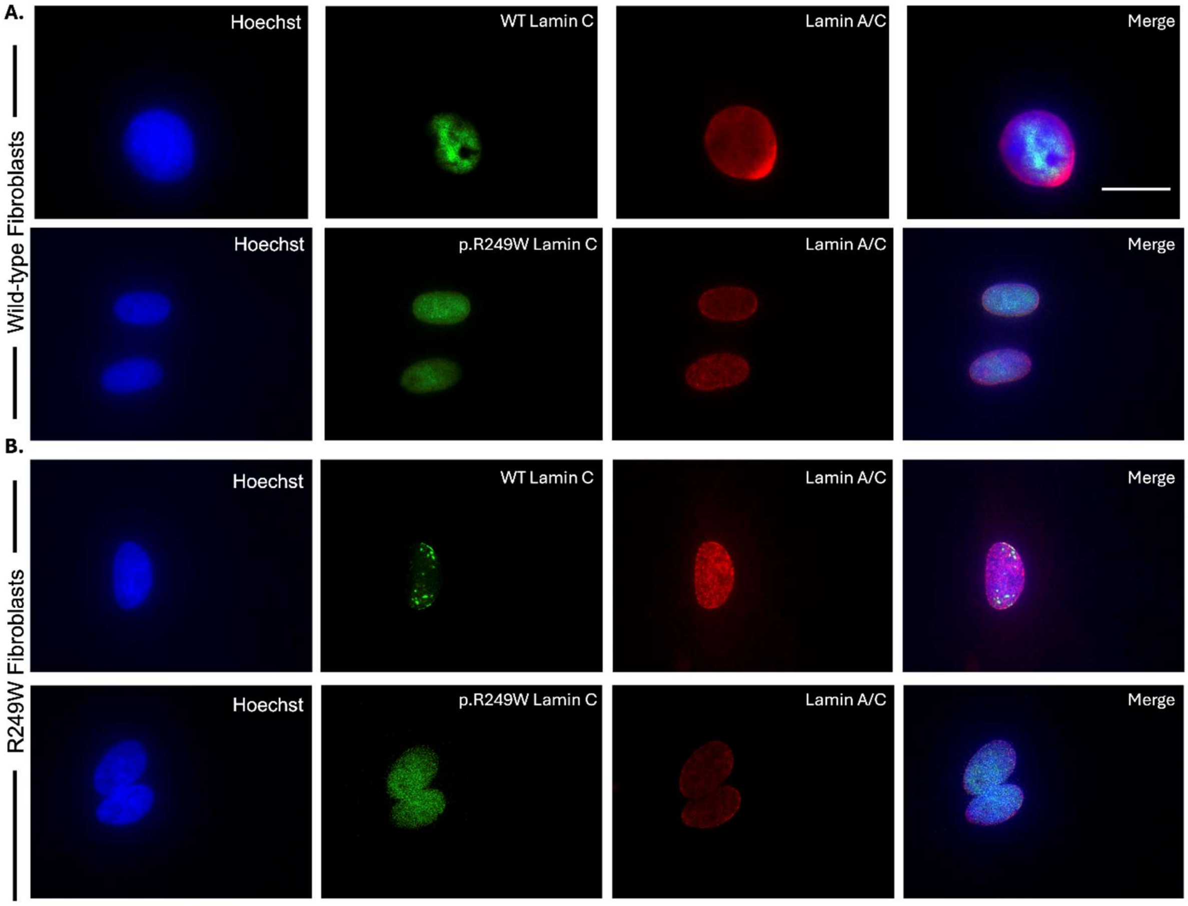

Figure S8: Transduction of p.R249W Lamin C is not sufficient to induce a polar localization phenotype. (**a**) Representative immunofluorescent images of wild-type fibroblasts transduced with either GFP-tagged wild-type Lamin C or p.R249W Lamin C. Immunostaining for overall Lamin A/C is shown in red. (**b**) Immunofluorescent images of p.R249W patient-derived fibroblasts transduced with wild-type Lamin C. Immunostaining for overall Lamin A/C is shown in red. Scale bar: 10 µm

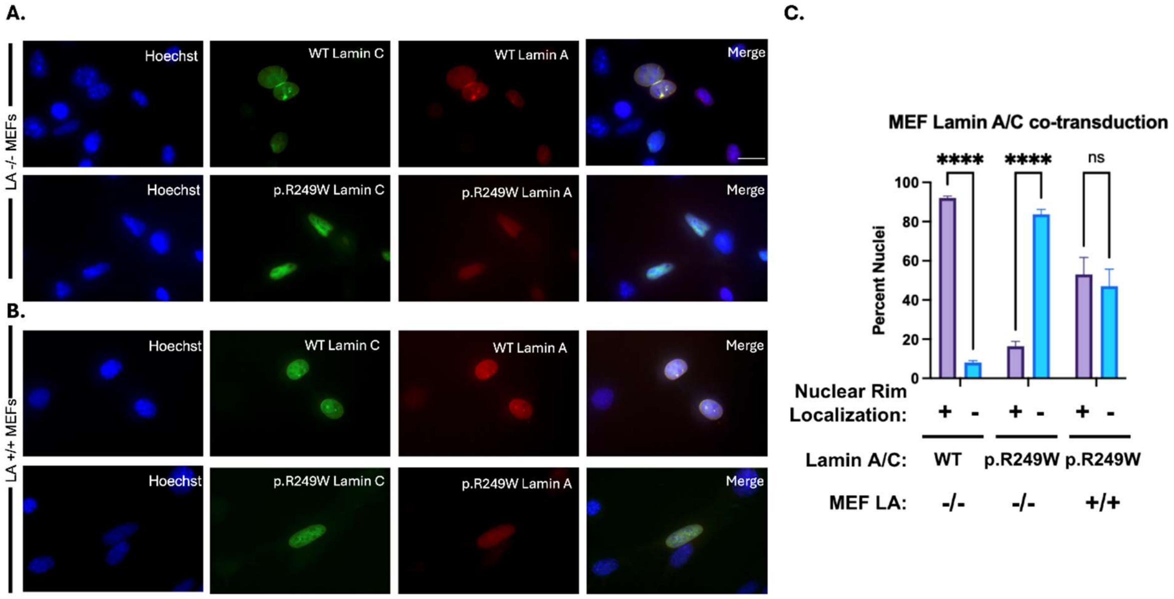

Figure S9: Representative fluorescence imaging of MEF transduction with either wild-type Lamin A/C or p.R249W Lamin A/C. (**a**) LA -/- MEFs or (**b**) LA +/+ MEFs were transduced with either wild-type Lamin A/C or p.R249W Lamin A/C. Hoechst stain (blue) represents nuclei. Scale bar: 10 µm. N=3 (**c**) Nuclei count of MEF LA -/- or MEF LA +/+ transduced with either wild-type Lamin A/C or R249W Lamin A/C. 100 nuclei were counted per replicate. N=3
